# Thermal Imaging to Assess the Health Status in Wildlife Animals under Human Care: Limitations and Perspectives

**DOI:** 10.3390/ani12243558

**Published:** 2022-12-15

**Authors:** Daniel Mota-Rojas, Alfredo M. F. Pereira, Julio Martínez-Burnes, Adriana Domínguez-Oliva, Patricia Mora-Medina, Alejandro Casas-Alvarado, Jennifer Rios-Sandoval, Ana de Mira Geraldo, Dehua Wang

**Affiliations:** 1 Neurophysiology, Behavior and Animal Welfare Assessment, Department of Agricultural and Animal Production, Universidad Autónoma Metropolitana (UAM) Unidad Xochimilco, Mexico City 04960, Mexico; 2 Mediterranean Institute for Agriculture, Environment and Development (MED), Institute for Advanced Studies and Research, Universidade de Évora, 7006-554 Évora, Portugal; 3 Facultad de Medicina Veterinaria y Zootecnia, Universidad Autónoma de Tamaulipas, Victoria City 87000, Mexico; 4 Facultad de Estudios Superiores Cuautitlán, Universidad Nacional Autónoma de México (UNAM), Cuautitlan Izcalli 54714, Mexico; 5 School of Life Sciences, Shandong University, Qingdao 266237, China

**Keywords:** infrared thermography, thermal status, pain, thermal window

## Abstract

**Simple Summary:**

Ensuring the welfare of wildlife under human care requires tools to monitor their health and well-being. Infrared thermography is a non-invasive technique for assessing thermal states that measure the radiation emitted from the skin in distinct anatomical areas, known as thermal windows—anatomical regions with abundant capillaries and arteriovenous anastomosis that facilitate heat exchange with the environment. However, thermal windows for wildlife species have not yet been established due to the different characteristics of their skin, coats, fur, or coloring. This review discusses published information on the usefulness of the ocular, nasal, thoracic, abdominal, and podal anatomical regions as thermal windows for evaluating these animals’ thermal responses and health status and monitoring habitat design. Another aspect that must be considered for wildlife under human care is the limitations of distinct species due to differences between animals and critical factors. Future studies should focus on establishing a precise application for each thermal window according to the specific characteristics of distinct animal species.

**Abstract:**

Promoting animal welfare in wildlife species under human care requires the implementation of techniques for continuously monitoring their health. Infrared thermography is a non-invasive tool that uses the radiation emitted from the skin of animals to assess their thermal state. However, there are no established thermal windows in wildlife species because factors such as the thickness or color of the skin, type/length of coat, or presence of fur can influence the readings taken to obtain objective, sensitive values. Therefore, this review aims to discuss the usefulness and application of the ocular, nasal, thoracic, abdominal, and podal anatomical regions as thermal windows for evaluating zoo animals’ thermal response and health status. A literature search of the Web of Science, Science Direct, and PubMed databases was performed to identify relevant studies that used IRT with wild species as a complementary diagnostic tool. Implementing IRT in zoos or conservation centers could also serve as a method for determining and monitoring optimal habitat designs to meet the needs of specific animals. In addition, we analyze the limitations of using IRT with various wildlife species under human care to understand better the differences among animals and the factors that must be considered when using infrared thermography.

## 1. Introduction

Infrared thermography (IRT) is considered a non-invasive, real-time technique used as a complementary diagnostic tool for several physiological and pathological processes in domestic and wildlife species under human care [1,2,3,4,5]. The control of heat exchange between body surfaces and the external environment plays a crucial role in regulating body temperature during different physiological phases and/or activities throughout the lifetime of homeotherms. Thermoregulatory adjustments can be induced by changes in environmental temperature and numerous physiological factors including age, fasting and food intake, stressful circumstances, and inflammation status, which can cause changes in internal temperature that result in variations in body surface temperature. The evaluation of surface temperature using infrared thermography—a non-invasive technique that captures images of a specific body region at a distance—represents a valuable tool for monitoring the animals’ physiological status, welfare, and stress responses [6,7]. The reactions of animals under stressful conditions primarily involve activating the sympathetic system and hypothalamic–hypophysis–adrenal axis (HPA) through the release of effector hormones, such as catecholamines, and glucocorticoid production, respectively. One result of this activation is stress-induced hyperthermia, which consists of an increased core body temperature with consequent temperature changes [8].

**Figure 1:**
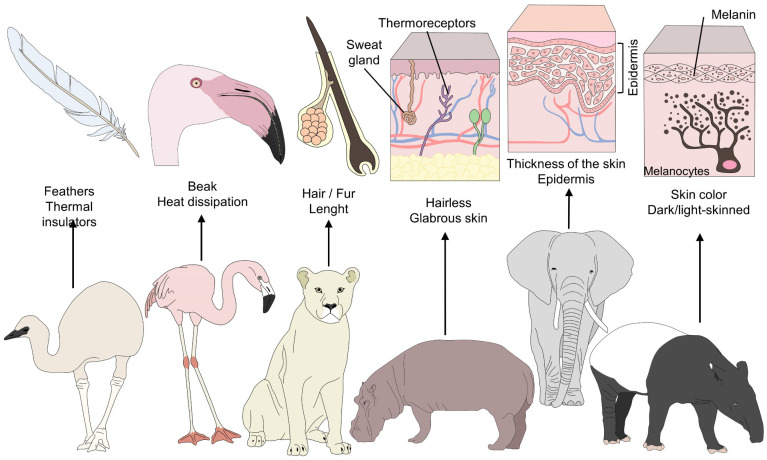
Morphological differences among furred, feathered, and bare-skinned animals. Thermoregulatory strategies in animals depend on various morphological elements. For example feathers serve as a thermal insulator in birds, and beaks facilitate heat dissipation. In contrast, the presence of hair and piloerection form a layer that conserves warm air to prevent any sudden drop in temperature. A thick layer of hair and dark hair color conserve heat and reduce radiation. Finally, skin color and the thickness of glabrous skin influence the amount of heat radiation in different species.

As a non-invasive tool, remote evaluation is considered advantageous because it minimizes handling or the use of chemical restraints [9] and prevents the development of processes like stress hyperthermia in small rodents and birds [10]. However, the wide diversity of species under human care has distinct anatomical, morphological, and physiological traits. For example, feathers are thermal insulators that maintain temperatures within normal ranges, but their presence limits the use of IRT, which requires zones with bare skin. Therefore, the eye region and legs are the preferred thermal windows in birds for evaluating superficial temperature changes [10]. In the case of animals with sparse hair, such as elephants, rhinoceroses, and hippopotami, or those that do not have long hair, like giraffes, or equids like zebras, IRT can be used in the abdomen, thorax, or ears. In contrast, for carnivores or species with winter coats, the recommendation is to evaluate windows located in the joints, feet (on the caudal side), or facial region (Figure 1) [1,11].

In addition to the features mentioned above, factors both external (environmental temperature, capture angle, distance to the target species, wind speed, and humidity) and internal (physiological state, feeding, metabolism, etc.) must also be considered to determine the validity and reliability of each anatomical region described as a potential thermal window. Therefore, the aim of this review, is to discuss the usefulness and application of the ocular, nasal, thoracic, abdominal, and podal regions as thermal windows to assess thermal responses and health status in animals. The limitations of IRT are also analyzed to better understand the species-specific characteristics that must be considered when using IRT with wild animals that are under human care. A literature search was performed to develop this review using the Web of Science, Science Direct, and PubMed databases to identify recent studies on the applicability of IRT with wild animals. The search keywords included “infrared thermography”, “zoo animals”, “wildlife under human care”, “zoo animal disease”, “zoo animal inflammation”, and “non-invasive zoo animal health”. The inclusion criteria were studies that used IRT as a complementary tool for diagnosing inflammatory, physiological, and/or pathological processes by evaluating thermal states in wildlife species. No year limit was placed on inclusion.

## 2. Advantages of IRT for Assessing the Thermal Status of Zoo Animals

**Figure 2:**
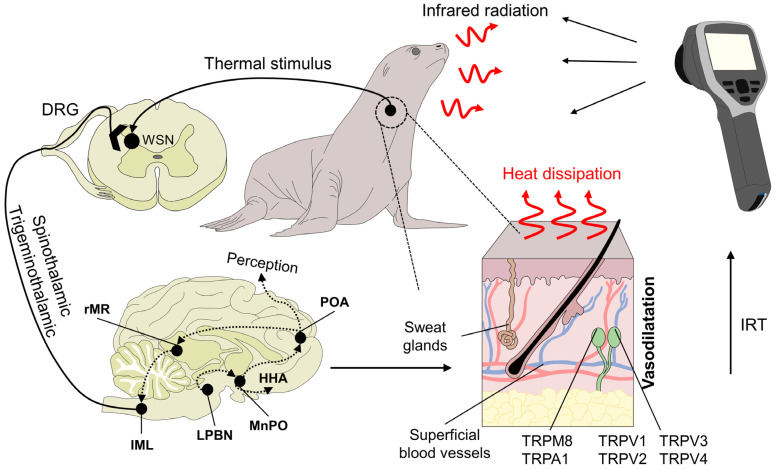
Physiological basis of infrared thermography and mechanisms of heat loss. Peripheral receptors in the dermal tissue, such as transient receptor potential vanilloid (TRPV, TRPV1, TRPV2, TRPV3, and TRPV4) or melastatin-related transient receptors (TRPM8), respond to heat and cold environmental stimuli, respectively. TRP ankyrin (A1) is postulated to have a function in noxious cold sensation and mechanosensation. These receptors initiate the transduction of the stimulus through primary peripheral nerve endings connected to secondary neurons in the spinal cord. Upon reaching this structure, warm-sensitive neurons (WSN) are activated and project the signal along the spinothalamic and trigeminothalamic pathways. Both of these tracts are connected to higher brain structures, such as the lateral parabrachial nucleus (LPBN) and median preoptic nucleus (MnPO) in the preoptic area of the hypothalamus (POA). DRG: dorsal root ganglion. HHA: hypothalamo-hypophyseal-adrenocortical. IRT: Infrared thermography. Their role is to generate behavioral, autonomic, and neuroendocrine responses. One of the autonomic responses to heat stimuli is peripheral vasodilatation by sympatho-adrenergic neurons in the rostral medullary raphe (rMR) and the intermediolateral nucleus of the spinal cord (IML), which increases heat dissipation. This response is captured by the lens of the infrared thermographic camera to generate a radiometric image.

IRT uses physical laws and surface properties to determine the superficial temperature of animals by measuring the amount of infrared radiation they emit (Figure 2) [12]. One of the main advantages that IRT offers is real-time, non-contact measuring of the surface temperatures of animals from distances of less than 1 m to over 1000 m, depending on species and the specific anatomical region selected [13]. This means that animals do not require extensive handling, physical restraint, or sedation, which is especially important when handling wild animals or those in zoos [1,14,15]. The IRT technique uses a camera with specialized thermal imaging lenses with diamond or germanium optical components and film to capture infrared radiation. A sensor processes this radiation to produce a digital radiometric image converted into a color pattern reflecting the temperatures detected [16].

The use of IRT with wild animals has focused primarily on detecting inflammatory processes, but other applications include aspects of thermoregulatory physiology in different taxa, population counts and comparisons, and evaluating the stages of pregnancy. IRT is helpful in the case of pregnancy because increased blood irrigation causes an increase in the surface temperature at the level of the flank in pregnant females. Additionally, it can help determine the association of certain emotional states with temperature changes and indirectly evaluate the habitat or housing of those animals [11,17].

**Figure 3:**
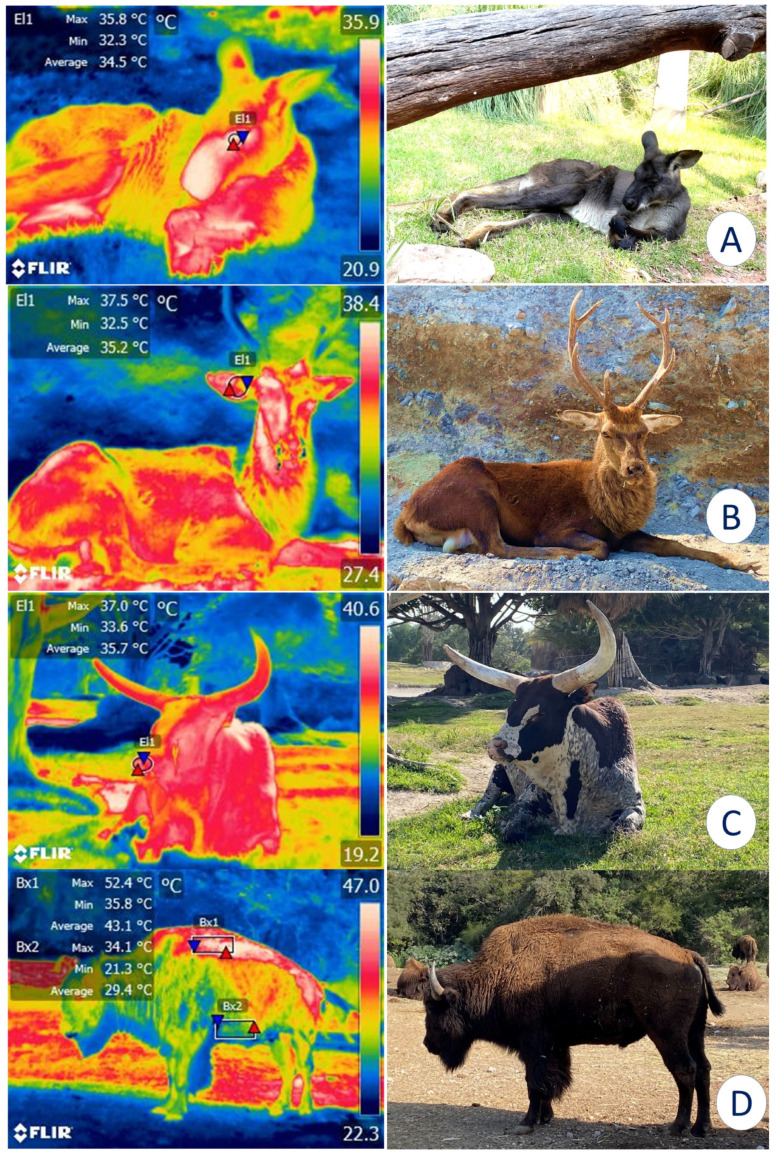
Importance of natural or artificial shade for the thermoregulatory behavior of various species. (**A**). Although this kangaroo (*Osphranter rufus)* is resting under natural shade, the average temperature of the lacrimal caruncle (El1) is 34.5 °C. (**B**). A sika deer (*Cervus nippon*) under shade shows an average temperature of 35.2 °C in the auricular region (El1). (**C**). The average nasal temperature of 35.7 °C of an ankole-watusi (*Bos taurus taurus ankole*) under the shade of a tree is depicted. (**D**). In an American bison (*Bison bison*), a comparison between the dorsal (Bx1) and ventral (Bx2) regions can be observed. An average temperature difference of 13.7 °C was registered between them. Therefore, shaded areas are a key element for ensuring the thermal comfort of wild animals under human care (Radiometric images and photographs by the authors). Maximum temperature (red triangle), and minimum (blue triangle). Thermal images obtained using a FLIR thermal camera.

For these reasons, IRT has been proposed as a method for monitoring enclosures and their characteristics to determine whether they comply with legal regulations and satisfy the basic biological needs of the species (e.g., shaded areas, bodies of water, adequate water supply) [18]. For example, alterations in the surface temperatures of elephants have been reported when the micro- or macroclimate of the installations are inappropriate for this species [19]. Similarly, in bears [20] and Bengal tigers (*Panthera tigris*) [21], IRT has recorded surface cutaneous temperature values and associated them with certain thermoregulatory behaviors, such as piloerection, seeking refuge in shaded areas, or rolling in the dirt to achieve thermoneutrality. This technique has also been used to assess the characteristics of the housing provided, which may favor or inhibit thermal comfort. Figure 3 shows the importance of providing sources of natural shade in enclosures for wildlife under human care since direct solar radiation without a proper resting area can cause heat stress in zoo animals.

Concerning thermal physiology, IRT recognizes several thermal windows that can be used for different species, including the African elephant (*Loxodonta africana*) [22], otters (*Lutra lutra* and *Pteronura brasiliensis*) [23], mole rats (*Fukomys mechowii* and *Heliophobius argenteocinereus*) [24], ground hornbills (*Bucorvidae*) [25], and koalas (*Phascolarctos cinereus*) [26]. Clinically, changes in thermal patterns, such as abnormal or asymmetrical thermal distribution, have been associated with pathologies, alterations in blood circulation, and inflammatory processes. In this area of study, Hilsberg-Merz [27] used IRT to evaluate problems affecting the joints and feet of elephants, rhinoceroses, giraffes, and hippopotami using IRT, while Church et al. [13] diagnosed a respiratory condition in an elephant related to a poorly positioned molar that was affecting the sinus area. In another study, Hurley-Sanders et al. [28] utilized IRT with fluorescein angiography to confirm a compromised vascular system and injury to the right wing in a Chilean flamingo (*Phoenicopterus chilensis*) that had a clinical history of acute weakness in that limb.

Thermography has also been used for the early diagnosis of diseases such as rabies in raccoons [29] and foot-and-mouth disease in mule deer (*Odocoileus hemionus*) [30]. Hurley-Sanders et al. [31] used it to monitor the healing process of an injury to the left forelimb of Gregory’s wolf (*Canis rufus gregoryi*) after a skin graft. Results showed that IRT could aid in the early detection of a poor blood supply to the graft based on the stability of the vascularization of the transplanted skin. Recently, IRT has begun to be used in a field of research where emotional states derived from various stimuli have been found to correlate with thermal responses, especially in primates like rhesus monkeys (*Macaca mulatta*) [32], gorillas [33] and chimpanzees [34].

However, applying the IRT technique more widely in medical attention for zoo animals and wildlife species under human care requires not only a deep understanding of the anatomy of a wide range of species but also and evaluation of numerous other elements: the fact that humans do not have absolute control over the animal (movement, position relative to the sun, body zones, etc.), and environmental factors (e.g., direct radiation, humidity, rain, wind) that impact the accuracy and interpretation of the temperature readings obtained [1]. Specific body regions have been proposed for using IRT with individual species considering their characteristics and challenges and each one’s morphological properties.

## 3. Facial Windows

### 3.1. Ocular Window (Regio Ocularis)

**Figure 4:**
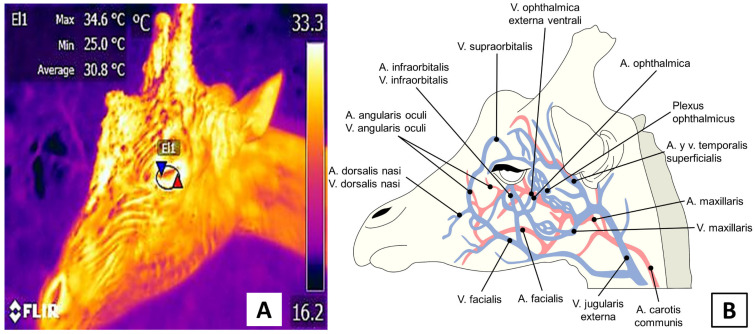
Depiction of the ocular window of a giraffe (*Giraffa camelopardis*). (**A**). A circle, approximately 3 cm in diameter encompasses the periocular region and a small portion of the eyelid. (**B**). Blood irrigation in this window comes from the supraorbital and infraorbital arteries, whose sympathetic innervation proceeds from branches of the facial nerve.

The ocular window assesses circulatory changes and thermal responses in the eye and the proximal area. Its usefulness has been reported in bovines [35], pigs [36], and dogs [37]. This window is surrounded by abundant periocular capillaries branching from the supra and infraorbital arteries. The latter irrigates the lacrimal canaliculi. Because its capillaries have autonomic innervation, they are sensitive to autonomic activation, especially during periods of acute stress. The area of the ocular window and its irrigation are represented in Figure 4.

The lacrimal caruncleis known to have sympathetic-innervated blood vessels that can respond to the activation of the HPA when exposed to a stressor. This is due to the secretion of catecholamines and the vasoconstriction response by its capillaries [38]. This effect has been reported in domesticated species such as sheep [2] and horses [39] during handling and transport, respectively. In those events, the temperature of the lacrimal caruncle has been seen to increase by up to 1 °C. In the case of wildlife species, Narayan et al. [26] evaluated the temperature of the lacrimal caruncle, abdomen, ears, and paws of three koalas born in captivity. They observed that the surface temperature of the ocular region had the greatest consistency and showed a significant increase compared to the ear region (31.6 °C vs. 23 °C, respectively), with a coefficient of variation of 4.83% in contrast to the values obtained from the ear (19.68%). This shows the window’s usefulness for evaluating the body temperatures of animals. Their conclusions are similar to the findings of Melero et al. [40], who compared the thermal windows of five species of marine mammals: two cetaceans, a beluga whale (*Delphinapterus leucas*) and bottlenose dolphin (*Tursiops truncatus*), and three pinnipeds, a Patagonian sea lion (*Otaria flavescens*), a harbor seal (*Phoca vitulina*), and a Pacific walrus (*Odobenus rosmarus divergens*). The ocular region was considered appropriate for determining body temperature in the pinnipeds, the ocular region was considered appropriate for determining body temperature. In contrast, in the cetaceans, the internal mucosal region of the open blowhole was suggested as the most stable thermal window for monitoring body temperature non-invasively, as it showed differences of 1 °C compared to the normal rectal temperature of these species.

The studies described above are associated with the clinical usefulness of thermography focused on the ocular region for determining the thermal status of animals, which can be confirmed by similarity with the values obtained by conventional methods, such as rectal temperature. However, the effectiveness of IRT has also been compared to other approaches. Stryker’s [21], for example, compared the thermal response of the eye to standard methods (rectal and axillary readings) and thermoregulatory behaviors in four species of large felines, namely, lions (*Panthera leo*), jaguars (*P. onca*), pumas (*Puma concolor*), and snow leopards (*P. uncia*). Using IRT, he identified that the surface temperature of the eye was similar to rectal values (39.9 °C and 39 °C, respectively). A second important observation was that the warm-climate felines (lions, jaguars) spent more time lying down (40–84%) than the cold-climate species (pumas, snow leopards) and had relatively less time performing active behaviors (5–10%). That study showed that temperature readings could provide information on the welfare of animals in enclosures when associated with different species’ typical behaviors for adapting to their environment.

A study by South [41] reported a similar response in the European hedgehog (*Erinaceus europaeus*). There, IRT was applied to monitor hypothermia during hibernation using ocular temperature as a reference in 55 animals (23 males, 32 females). The temperature obtained with an axillary thermometer was significantly lower (1.3 ± 0.18 °C) than that of the periocular region (33.7 °C and 35.5 °C, respectively). Likewise, the ocular reading diagnosed hypothermia more precisely than relying on behavioral and clinical signs, suggesting that IRT is an accurate tool that can aid in evaluating the thermal states of animals and associate them with specific pathological and physiological conditions, thus contributing to the rescue of these species.

In contrast to the reliability and usefulness of IRT described above, a study of 39 Guianan squirrel monkeys (*Saimiri sciureus*) showed that facial temperature correlated poorly with rectal recordings (r^2^ = −0.10, 95% IC= −0.27–0.07), as the average difference between the methods was 3.4 °C [42]. These results show that the precision of IRT depends greatly on choosing an adequate region for recording surface temperatures. Similar to descriptions of domesticated species, ocular IRT readings can be used to determine stress levels [2,43]. A study of giraffes (*Giraffa camelopardis*) evaluated the thermal response of the ocular and auricular regions under the assumption that, for these animals, feeding could be a stressful event. Results showed a correlation between blood cortisol levels (*p* = 0.269) and temperature that did not change before or after feeding. A positive relationship was also observed between the eye’s and the ear’s thermal response (Spearman rank *p* = 0.759, *p* < 0.0001). The authors attributed the absence of any ocular thermal response to a potentially enjoyable emotional state, suggesting that IRT could be useful for identifying emotional states in animals [44]. Vaz et al. [45] found a similar response in African lions (*P. leo*), where the IRT response at the ocular level was used to understand large felines’ personality and stress physiology, when coordinated with ethological studies and fecal glucocorticoid levels. The authors identified three personality types by studying 22 African lions in two locations, (dominant, gentle, and neurotic). The animals had differences in their fecal cortisol metabolite levels, but these did not influence their thermal responses, so it was impossible to correlate body surface temperature with the personality type or age of the animals. One important element is the standardized distance of 1 m between the thermal camera and the ocular region. Another is lens angle, which must be perpendicular (90°) to the sagittal plane to ensure valid readings [46,47].

As observed in zoo species, unlike in domestic animals, measuring the surface temperature at the ocular level is a remote method to assess body temperature without handling the animals. However, its application to assess the stress degree or ANS activation has yet to be well established. Therefore, as reported in large ruminants, future studies must consider each species’ emotional factor and anatomical differences, as reported in large ruminants [38]. 

Finally, it is important to consider that the ocular window can be evaluated by other techniques, such as optical coherence tomography (OCT). This method could help evaluate in vivo vascular responses by identifying the reactions of surface ocular blood vessels. Meleppat et al. [48] used this approach with aerial, aquatic, and terrestrial species like the Long Evans rats, gray short-tailed opossums (*Monodelphis domestica*), white sturgeons (*Acipenser transmontanus*), and great horned owls (*Bubo virginianus*). OCT has proven effective in identifying retinal and choroidal vascular structures and retinal layers [49]. Therefore, it can be suggested as a novel tool for diagnosing ophthalmologic diseases since the responses recorded by IRT may differ when animals are exposed to stressors.

### 3.2. Auricular Window (Regio Auricularis)

**Figure 5:**
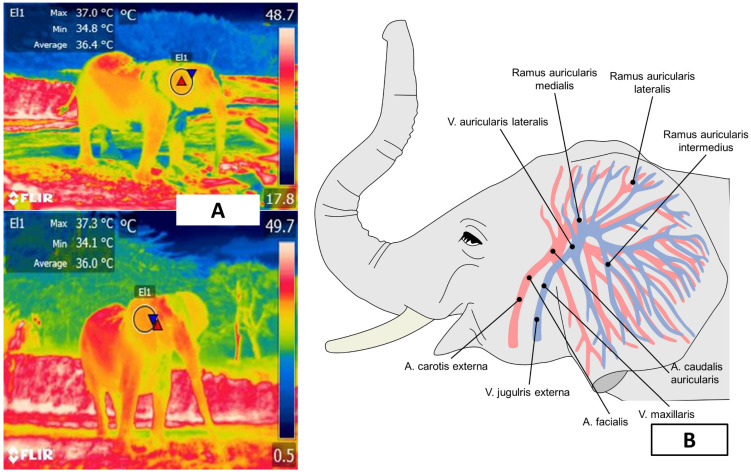
Auricular window of an African elephant (*Loxodonta africana*). (**A**). A circle around the external auditory canal represents the window. (**B**). The blood supply to the ear comes from the branches of the lateral auricular artery and the medial and marginal areas of the ear.

The auricular window is the most important region for thermographic evaluation in elephants. The ears of elephants represent approximately 20% of the total body surface area [50] and have a dense subcutaneous vasculature provided by the auricular arteries and veins (*arteria auricularis caudalis*) on the caudal surface [51] (Figure 5). The elephants’ ears dissipate as much as 8% of body heat [52], though Hilsberg-Merz [1] mentions that African elephants—the species with the largest ears—can lose up to 30% of excess heat by radiation and convection through the ears. In elephants, the extension of the auricular pavilion compensates for the lack of sweat glands [22].

Because of these characteristics, IRT can assess the auricular region to determine the thermostability of the species and the influence that environmental temperature and/or enclosure design exert on it. Phillips and Heath [50] studied the heat exchange mechanism in four female African elephants (*L. africana*), finding that surface temperatures of the pinna change in response to environmental temperature through vasomotor action. They further stated that readings proximal to the external auditory meatus were higher than those registered at the extremities of the pinna (39 °C and 18 °C, respectively, at an ambient temperature of 18 °C). Parallel to changes in auricular microcirculation, ear-flapping is another method of thermoregulation that is believed to transfer heat by rotational oscillations [53]. This process involves the rotator, *auriculo occipitalis*, *postauriculatis*, *platysma myoides*, and *sphinctor profundus* muscles in the Loxodonta genus, and the rotator, *auriculo occipitalis*, *postauricularis*, and *zygomatico auricularis* muscles in the Elephas genus [54]. Therefore, the vasculature and motor control of the ears allow heat dissipation through radiative heat and convection, facilitate heat exchange in that region, and are considered a useful window for evaluating the thermal comfort zone of these species. Ear-flapping dissipates heat by forced convection, thus increasing the rate of heat loss.

A study of six African elephants (*L. africana*) by Weissenbock et al. [22] reported similar results. They took 325 thermograms of six body regions at times of 5, 10, and 30 m: head, proboscis, torso, forelimb, hindlimb, and ear. The ear lobe and distal areas of the pinna delimited the latter. IRT in this region increased in a range of 33.8–35.3 °C in warm weather after the animals returned to their indoor enclosure. Seventeen minutes after returning, the average ear temperature was 17.1 °C, while after 68 min it increased, revealing areas of maximum and minimum temperatures at 33 °C and 22.2 °C [22]. This area remained the body region with the lowest surface temperature. Intriguingly, the authors recorded different thermal patterns in the two ears of the same animal. This difference has also been reported in leporids, where sympathetic innervation can control the temperature of each pinna independently. In contrast to the variability and absence of any established pattern in other thermal windows, auricular IRT associates the thermoregulatory response of the animals with their housing conditions. Therefore, it is essential to (i) provide water for frequent bathing and (ii) consider other morphological characteristics, such as the elephant’s wrinkled skin, which increases the surface area and ensures more efficient heat loss due to water evaporation [50].

**Figure 6:**
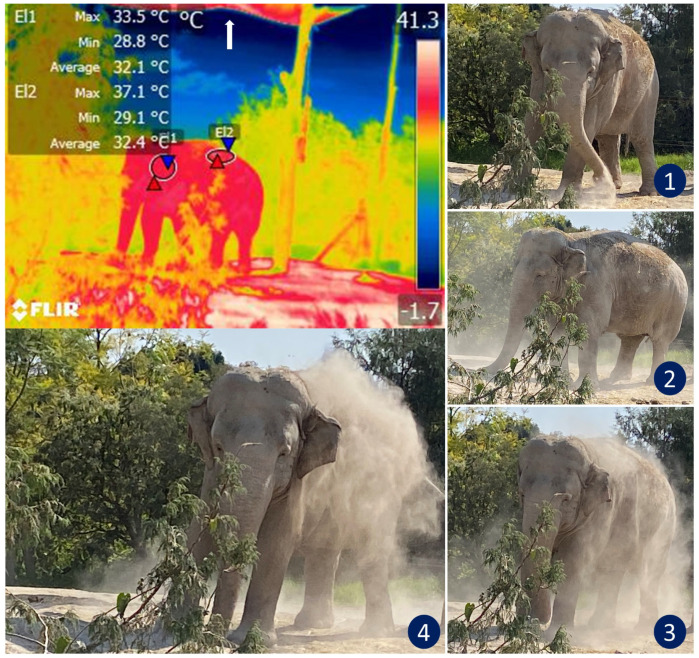
Thermoregulatory behavior in an African elephant (*Loxodonta africana*) under heat stress. The sequence of digital images shows dustbathing in an elephant exposed to high temperatures that triggered thermal imbalance (see images 1, 2, 3, and 4). The radiometric image shows the average temperature of the auricular (El1) and dorsal (El2) thermal windows. Under normal conditions, the ear temperature in elephants oscillates between 25 and 31 °C. However, the thermal image shows a temperature in the auricular region as high as 33.5 °C. IRT imaging reveals that although the animal covers its back with dust to thermoregulate, this behavior might be insufficient to achieve thermostability in this environment because the maximum temperature of the dorsum was 37.1 °C. Although the enclosure has a shade mesh as a roof to reduce direct sunlight (white arrow), it is not enough to reduce thermal stress in this species. (Radiometric images and photographs by the authors).

The design of the enclosures also influences thermostability. Here, IRT can help evaluate the thermal responses of animals. Figure 6 illustrates how this thermal window was used to assess the thermal states of African elephants exposed to heat stress as they performed natural thermoregulating behaviors, such as ear-flapping, bathing and splashing in mud. The authors of a study of Asian elephants that had recently been moved to a new enclosure observed lethargy in all subjects. Their IRT temperature readings of the ears were similar to those recorded for the body. They considered this an abnormal pattern since the ears of elephants are structures designed for heat dissipation. However, upon analyzing the macro environment of the pachyderms, the authors found high levels of humidity (95%) and insufficient access to sources of water, two conditions that resulted in heat stress and higher ear temperatures as compensatory mechanisms [19]. In that case, the vapor partial pressure was close to the saturation pressure, thus reducing the evaporation rate and decreasing evaporative heat loss. The only possible response by those animals was to increase the peripheral temperature (increased auricular IRT) to maximize heat loss through radiation and convection. An important point to emphasize in that study is that the entire group showed the same pattern of increased auricular IRT. This made it possible to non-invasively discern between a pathological process in a single individual and a general, simultaneous response by the herd [1].

IRT has also been utilized to diagnose inflammatory pathologies in Asian elephants (*E. maximus*). Avni-Magen et al. [55] found that assessing regions like the head, ears, body, and limbs at a distance of 2–3 m identified inflammatory processes with a sensitivity and specificity of 89.2 and 83.4%, respectively, and a negative predictive value (NPPV) of 99.4%. In that study, increases of up to 2° in the delta temperature of the ear surface made it possible to detect inflammatory processes before clinical diagnoses were made due to visible signs. However, due to the low positive predictive value (PPV) of 19.3%, the authors concluded that IRT could be recommended as a complementary method that requires other confirmatory diagnostic techniques.

The effects of pathologies and thermostability have been studied in other zoo species, such as equids, large felines, large ruminants, and wild birds, among others. Da Costa et al. [56] used IRT with 18 captive canines, including the crab-eating fox (*Cerdocyon thous*), maned wolf (*Chrysocyon brachyurus*), and hoary fox (*Pseudalopex vetulus*) in a study that lasted almost a year. Readings showed that the temperature of the left ear increased, compared to the right ear (24.6 and 21.3 °C, respectively). This effect was attributed to an abscess that was not perceivable in a remote inspection. In addition, it was also possible to verify post-treatment efficacy since the thermographic values were equal in both ears after 9 days of treatment. A study of giant sable antelopes (*Hippotragus niger*) and mishmi takins (*Budorcas taxicolor taxicolor*) used thermography to evaluate the effects of heat stress and how it influenced the animals’ behavior and thermoregulation mechanisms [1]. In contrast, in a study of a feline patient (*Leopardus tigrinus*) with renal failure, IRT did not show diagnostic validity or reveal any abnormal thermographic patterns in the animal’s pads due to the thickness of the dermis, which impeded taking accurate infrared reading [56]. Similarly, the ear region of Koalas, unlike the studies of elephants mentioned above, proved to be the least consistent window (compared to the ocular, abdominal, dorsal, and paws), as it showed a coefficient variation of only 19.68% and an average temperature of 22.11 °C, as opposed to the ocular IRT readings, which had a coefficient variation of 4.83% [26].

Finally, the application of IRT with captive wildlife species could be adapted for use with animals living in the wild. For example, auricular IRT has been evaluated in chimpanzees concerning behavioral changes, such as whimpers and aggressive barks, to assess the effect of emotions in response to conspecific vocalizations. In those test animals, after listening to aggressive bark vocalizations, the auricular surface temperature increased (*p* < 0.05) by an average of 0.7 ± 0.25 °C, but the temperature increase was greater during neutral calls due to an increase in blood flow that improved the performance of the animals’ auditory sensitivity to the vocalizations of their conspecifics [57]. These data demonstrate the usefulness of the auricular/pinna region for assessing various animal processes, especially the limits of thermoneutrality within which species held in captivity must be maintained to avoid behavioral and physio-metabolic complications triggered by heat stress.

### 3.3. Nasal Window (Regio Nasalis)

**Figure 7:**
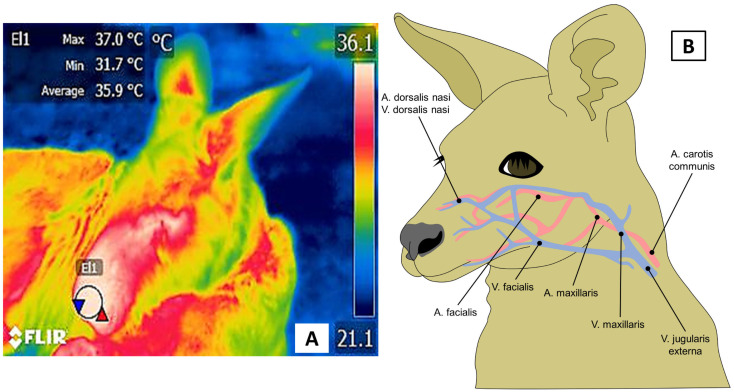
Nasal window in a kangaroo (*Osphranter rufus*). (**A**). A circle around the nasal region, including both nostrils, delimits the nasal window. (**B**). Circulation in the nose depends on branches of the maxillary artery and the *a. facialis* and *a.* and *v. dorsalis nasi*, which are innervated by the facial nerve.

The nasal window is an area of particular interest due to the sympathetic branch’s ability to reflect ANS activity during events involving stress, excitement, physical activity, and disease processes, all of which cause vasoconstriction of peripheral blood vessels [58,59]. Figure 7 shows the vasomotor action derived from branches of the maxillary and sphenopalatine arteries, which are innervated by the facial nerve [60].

Ross et al. [34] recorded nasal thermograms from 29 chimpanzees (*Pan troglodytes*) to identify the emotional arousal of those animals according to the species’ behavioral repertoire. In the 538 thermographic images taken in that study, the temperature of the nasal region decreased significantly in the individuals considered active compared to those classified as showing inactive behaviors. The authors concluded that the nasal region is a thermal window of great importance in this species but that needs to be evaluated or compared with other anatomical areas, such as the forehead, to achieve validation. In another study, the emotional responses and changes in cutaneous peripheral perfusion in the nasal area were assessed in 17 common marmoset monkeys (*Callithrix jacchus*) exposed to conditions with distinct emotional valence. The presence of food (considered an exciting event) increased nasal temperature by 0.8 ± 0.7 °C. In contrast, the presentation of negative stimuli led to a 0.5 ± 0.8 °C decrease in the temperature of this area. Decreased nasal IRT readings were also associated with aggressive vocalizations and piloerection during aversive events [61]. The physiological mechanism that explains this thermal response under exposure to a potentially aversive stimulus is sympathetic activation and the consequent secretion of catecholamines, which are responsible for triggering peripheral capillary vasoconstriction and, therefore, inducing a decrease in the temperature radiated at the surface level [16].

In other non-human primates, IRT has been applied to evaluate the thermal response of chimpanzees (*P. troglodytes*) to adverse auditory stimuli (vocalizations). According to the results, nasal temperatures decreased significantly (0.16 ± 0.18 °C, *p* < 0.001) compared to thermal responses in the ear canal—where the opposite effect was observed—as temperatures increased (0.06 ± 0.09 °C, *p* < 0.001) [57]. This same response was reported in a study of rhesus monkeys (Macaca mulatta), where a decrease in temperature at the nasal level (0.2 ± 0.1 °C) was observed when the animals were exposed to a threatening physical agent. In addition, and proportionally to the decrease in temperature, facial movements such as bared teeth, mouth opening, and constant lip licking were observed [32], all of which form part of the facial language this species employs to communicate mental states [32,62].

A study of gorillas (*Gorilla gorilla*) by Heintz et al. [33] assessed the temperature of the nasal region and its correlation with cortisol and oxytocin levels during training with positive reinforcement and cognitive tasks. Observations showed that the nasal temperature decreased significantly during both moments (*p* < 0.014), while salivary cortisol and oxytocin also decreased after the sessions. In contrast, a higher frequency of self-directed behaviors was observed during the controlled conditions. Those results show the inconsistency and difficulty of validating the nasal window as a method for evaluating emotions, though other authors consider it a reliable area for identifying emotional states in non-human primates under human care [63].

In contrast, McFarland et al. [64] evaluated thermal responses in different body regions of vervet monkeys (*Chlorocebus pygerythrus*), including the dorsum and face, especially the nasal region. Results revealed that although the temperature of the nasal region presented significant changes, they were not related to body temperature and thus could not be considered an effective approach to measure vasoconstriction accurately. The authors attributed this effect to the influence of environmental factors that can alter thermographic readings. Their observations were similar to those reported by Thompson et al. [65], who evaluated the nasal area of howler monkeys (*Alouatta palliata*). In that study, the nasal region was not found to reflect internal processes because autonomous processes regulate body temperature for the production and loss of heat. This observation allows us to understand why this window does not represent a relevant region for heat transfer in red-rumped agouti (*Dasyprocta leporina*), a species in which cooling due to water evaporation in the nasal mucosa can affect the temperature of this region [66].

**Figure 8:**
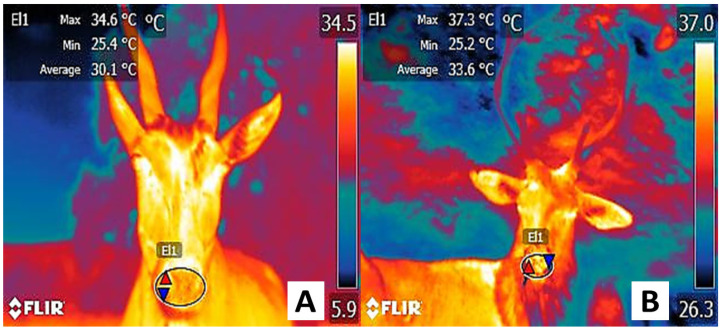
Comparison of the thermal state of the nasal region in healthy and sick animals. (**A**). Temperature of the nasal region in a gazelle (*Gazelle gazella*). This animal presents no health alterations or clinical signs, and its overall health condition is good, as shown by the maximum temperature (red triangle) of 34.6 °C in the nasal region (El1) and an average value of 30.1 °C. (**B**). Temperature of the nasal region in a deer (*Odocoileus virginianus*). In this case, an adynamic animal with upper respiratory disease and nasal discharge presents a maximum nasal temperature (El1) (red triangle) of 37.3 °C and an average temperature of 33.6 °C. Compared to the healthy animal, a difference between 2.7° and 3.5 °C can be seen. This may be due to a pathological process that includes the release of cytokines and prostaglandin E_2_, which can cause a feverish state and promote the activation of thermoregulation mechanisms such as vasodilation to effectuate heat loss.

Given these findings, it is evident that this thermal window can help identify and understand emotional states, especially in primates. Nevertheless, the contradictions and difficulties in associating microvascular vasomotor responses to a specific emotional event have made it necessary to explore its validity more widely and whether the response in primates could be found in other species. Without a doubt, the use of these thermal windows to evaluate the health status of zoo animals is a field that requires further studies to determine their usefulness. Preliminary results of the authors’ work with gazelles and deer, for example, have found that animals with clinical signs of respiratory disease have alterations in the surface temperature of the nose, as shown in Figure 8.

## 4. Body Windows

### 4.1. Thoracic Region (Regio Costalis) (Regio Pectoris)

Evidence from facial regions indicates that they can be used as thermal windows to determine physiological parameters such as body temperature and respiratory rate, which can help verify individuals’ health status [67,68,69]. One example is the case of water buffaloes, a species for which the thoracic window has been proposed to evaluate thermal comfort [70,71]. Body windows, like the thoracic region, have been mainly used to identify and follow up on inflammatory processes and infectious diseases, where IRT can assist by monitoring lesions and achieving timely diagnoses of hyperemia and hypothermia disorders [70,71].

The anatomical characteristics of this region and the network of blood vessels that supply muscles like the pectoral fascia (*fascia pectoralis*), ventral thoracic serratus (*serratus ventralis thoracis*), transversus thoracis (*transversus thoracis*), and thoracic rectum (*rectus thoracis*) during inflammatory or pathological processes generate the secretion of proinflammatory mediators, such as prostaglandins, serotonin, histamine, and interleukins. These substances cause local vasodilation of the capillaries that irrigate the muscles and, consequently, irradiate greater heat, which is detectable using IRT [72].

Menzel et al. [58] observed that the temperature in the thoracic region was 2.4 °C higher than in the abdominal region when determining the health status of the lungs of domesticated species such as pigs. This increase correlated positively with the thickness of the lung tissue layer and local temperature increases (r_S_ = 0.50, *p* = 0.03). In this context, a later study carried out with the same species related to febrile responses detected by IRT in animals inoculated with *Actinobacillus pleuropneumoniae* found a positive correlation between rectal and surface temperatures (r^2^= 0.52, *p* < 0.001), as the former was significantly higher than the latter (0.5 ± 0.3 °C vs. 0.3 ± 0.3 °C) (*p* < 0.0001), though this did not represent a meaningful difference (*p* > 0.05) [73]. Both of those studies showed the clinical capacity of IRT to detect local pulmonary inflammatory processes [74,75], though it performed poorly in identifying febrile states, as results were similar to those found in studies of the ocular thermal window.

What remains to be determined is the specificity of the thoracic region for detecting increases in surface temperature as a result of local pathological processes involving prostaglandins F2α (PGF2α) or interleukin-1 (IL-1) that cause inflammation of the surrounding lung tissue [76]. As da Costa et al. reported [56], IRT has been utilized in this area to evaluate injuries generated by trauma. Those authors analyzed the IRT of 42 felines and canines, including the maned wolf (*Chrysocyon brachyurus*), crab-eating fox (*Cerdocyon thous*), hoary fox (*Pseudalopex vetulus*), jaguar (*Panthera onca*), hoar puma (*Puma concolor*), and ocelot (*Leopardus pardalis*). In the dorsal region of the thorax and limbs, temperatures increased significantly by 3.1–3.3 °C, indicating an inflammatory process accompanied by clinical signs and confirmed by radiographic diagnoses. Although these findings helped detect conditions in the corresponding area, the authors reported that the density and thickness of the animals’ coats could affect thermal readings. The authors determined that because thicker coats trap more stable air, it is impossible to measure the radiation emitted by the epidermis accurately.

As reported by Hilsberg [1] in a review of IRT findings in different species of wild animals under human care, the thoracic window has been suggested for evaluating local flank lesions in Asian elephants (*E. maximus*) and white (*Ceratotherium simum*) and black rhinoceroses (*Rhinoceros unicornis*). In these animals, modifications of their daily activities were associated with lesions in the thoracic area of the right flank, making it possible to achieve early diagnoses with no need to immobilize the subjects. The author further stated that, unlike domestic species (such as the water buffalo and domestic cattle), the thorax region is not considered an effective window for determining thermal comfort, thus, this is a limitation that arises when validating this region [38].

Subsequently, the thoracic thermal window was proposed as a potential region that could assist in identifying pathological conditions, such as infectious diseases, and recognizing hyperthermia derived from the secretion of proinflammatory substances. Moreover, during the development of lesions in this area, vasodilatation and increases in local temperature allow the non-invasive detection of possible affected areas and, based on the information obtained by IRT, implement medical imaging or laboratory techniques to confirm diagnoses. However, species-specific factors, such as hair density and color, must be considered when using or validating this window because they can affect readings.

### 4.2. Abdominal (Regio Abdominis)

**Figure 9:**
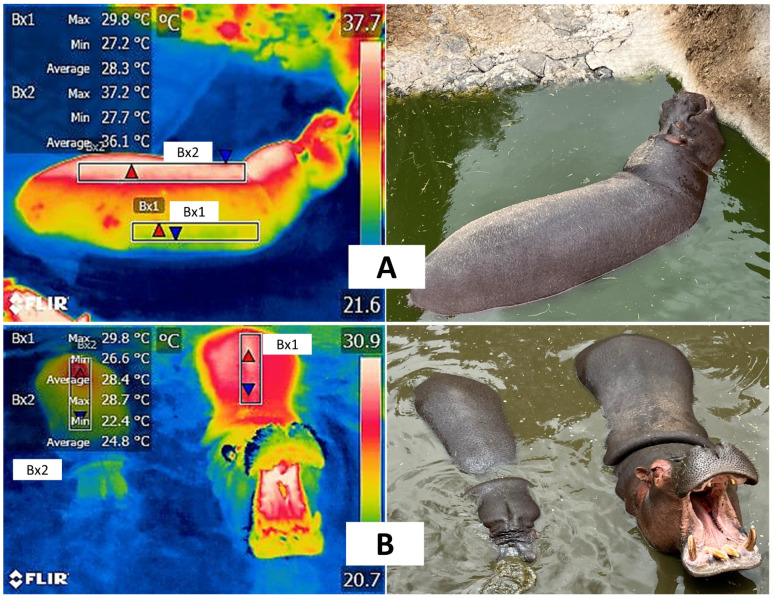
Importance of ponds or pools to improve the comfort level of hippopotami housed in zoos. (**A**). Dorsal and lateral body window with total immersion in a pond. A difference of 7.8 °C can be seen between the mean temperatures of the lateral (Bx1) and dorsal regions (Bx2). This is due to the effect of solar radiation that falls directly on the dorsal region, which, in contact with the lateral region with the water, can dissipate heat more efficiently and maintain lower temperatures. (**B**). Effect of immersion in water on the thermoregulation of a mother and her calf. A decrease of 3.6° °C is observed in the mean temperature in the offspring’s dorsal region (Bx2) in contrast to that of the mother (Bx1). This temperature drop is associated with the fact that the calf’s skin was moist when the image was taken. Both cases reflect the importance of ponds or pools to help maintain thermoneutrality in animals with poor thermoregulation mechanisms, like the hippopotamus (*Hippopotamus amphibius*). (Radiometric images and photographs by the authors).

The abdominal region of zoo animals is used less frequently as a window than previously mentioned regions. However, the irrigation that flows from the caudal aorta artery (*arteria aorta abdominalis*) and its branches to the abdominal organs or the dermal areas of the *regio abdominis cranialis*, *medius*, and *caudalis* quadrants provide the anatomical basis for evaluating physiological states such as gestation in some species [60] (Figure 9).

In rhinoceroses, IRT has detected increases in *regio abdominis medius* and *regio abdominis lateralis* temperatures from the end of the third trimester of gestation, with increases of up to 6 °C in the uterine zone compared to maternal body temperatures and decreases in the temperature of this zone (of approximately 2.6 °C) after giving birth [9]. Similarly, in a babirusa (*Babyrousa babyrussa*) housed at the Wilhelma Botanical and Zoological Garden, IRT was utilized as a complementary technique during gestation from August 2016 to January 2017. Krueger et al. [77] delimited a thermal window as a rectangular area enclosing the hindleg, and foreleg, spine, abdomen, and teats. The maximum temperatures of the right and left side of that thermal window correlated with the weeks of gestation (R = 0.66, R = 0.61, *p* < 0.05, respectively), with changes detected from the third trimester, but no differences between the two sides (*p* > 0.05). More specifically, the flank temperature reached 29 °C five days before parturition, and teat temperature increased from 27 °C in the fourth week of gestation to 32 °C five days before calving. This increase in the irradiated temperature of the teats was more evident than in other areas of the thermal window because the teats have glabrous skin due to an increase in energy demand for lactation. The authors also mentioned that one advantage of this window is that prolonged exercise or activity does not affect thermographic readings and, therefore, does not interfere with interpretations [9].

As has occurred in domesticated animals such as horses, in which pregnant mares show increases of 2 °C in the abdominal region compared to non-pregnant females [78], in larger zoo species, such as the white (*Ceratotherium simum*) and black rhinoceroses (*Diceros bicornis*), giraffe, and elephant, gestation can be diagnosed by superficial dermal changes during the first trimester [1]. Furthermore, IRT is suggested for use with rhinoceroses as a tool for detecting inflammatory processes and temperature alterations in animals with dermal pathologies [79].

Another application of IRT in the abdominal region is in evaluating the thermal needs of species such as big captive cats, including lions (*P. leo*), tigers (*P. tigris*), jaguars (*P. onca*), amur tigers (*P. tigris altaica*), pumas (*P. concolor*), and snow leopards (*P. uncia*). In these cases, IRT has been used to determine the influence of the conditions of captivity and the microenvironment on the animals’ thermoregulatory behavior [21]. Thermography was also used to observe the functionality and effectiveness of heat dissipation behaviors in nine Bengal tigers (*Panthera tigris tigris*), especially lying in a dorsal decubitus posture with exposure to the loins. Although this behavior was not prevalent in felines (between <4 and 13.1%), the surface temperature of the area increased as a vasomotor mechanism to dissipate heat during hot weather. This finding led the author to suggest the *regio pubica* as a promising field of study and recommended determining its validity as a thermal window and thermal threshold in this species. In addition, that study mentions the importance of considering key design factors of the installations, such as the use of glass in exhibits, which reflects solar radiation and can heat the enclosure due to short- and long-wavelength radiation, thus impacting the thermal comfort of captive animals [80]. In other studies, conducted with wild felines such as the jaguar (*Panthera onca*), jaguarundi (*Herpailurus yagouaroundi*), and oncilla (*Leopardus tigrinus*), IRT has been applied to detect conditions such as dermatitis, pododermatitis, and periodontitis. The authors of those reports affirm that IRT has a high sensitivity for evaluating facial (*faciei regions*) and appendicular regions (*membri thoracici and membri pelvini regions*), but low diagnostic sensitivity in regions such as the trunk (*regio pectoris*) and abdomen (*regio abdominis*), due to the presence of thick fur that interferes with thermographic readings [56]. Other windows suggested for these species are at the facial level (eye, mouth, tongue) [11]. They could help assess thermoregulation in wild felines [13].

In contrast to previous reports on koalas (*Phascolarctos cinereus*), Narayan et al. [26] studied the body regions of three captive individuals to determine the most suitable thermal window. In addition to the eyes, one window they could validate, the abdominal area had the highest consistency for measuring surface temperatures, with a coefficient variation of 11.01%, an average temperature of 22.13 °C, and visibility of 48.54% in radiometric images. This can be suggested as an option for this marsupial and other zoo animals when the aim is to evaluate their heat exchange efficiency, as well as for physiological states such as gestation, during which higher estrogen levels promote increases in irradiated temperatures, thus facilitating identification by IRT.

## 5. Podal Windows (Regio Carpi, Regio Tarsi)

**Figure 10:**
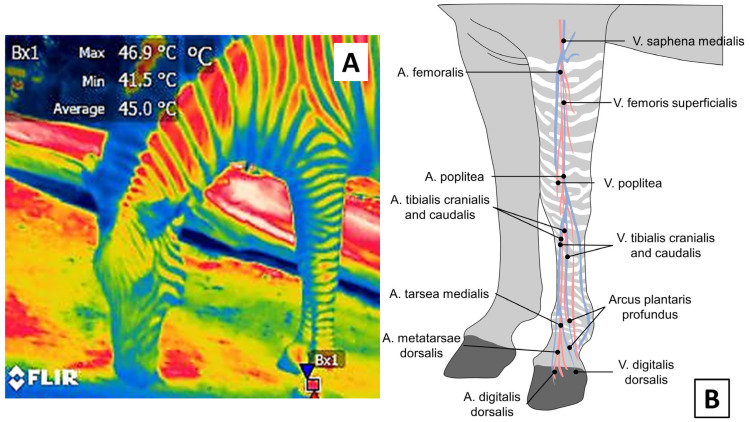
Podal window. (**A**). The podal region assessed using a rectangle that proximally encompasses the metacarpophalangeal joint down to the distal phalanx. (**B**). This window has been used to detect inflammatory processes in paws that generate the secretion of pro-inflammatory substances and, consequently, greater heat radiation.

The podal or phalangeal thermal window has been utilized mainly for joint pathologies or inflammatory injuries. Figure 10 shows the irrigation of this region by arteries such as the dorsal metatarsal (*metatarsea dorsalis*), medial tarsal (*tarsea medialis*), and *tarsea perforans* in the case of the pelvic limb, or the dorsal carpal (*carpeus palmaris*) and palmar (*palmaris*) arterial branches in the case of the thoracic limb [60]. When an inflammatory process develops due, for example, to the presence of bacteria, immune cells (neutrophils or macrophages) trigger the release of proinflammatory substances (e.g., serotonin, histamine, prostaglandin, interleukins) that cause local hyperemia [81], an effect that can be detected by IRT [1,27].

In large herbivores such as giraffes, rhinoceroses, elephants, and hippopotami, it is often difficult to observe the exact location of injured areas on limbs, especially when no clinical signs are visible [27]. In these cases, IRT can aid in identifying injured areas [19]. Dunbar et al. [29] evaluated changes in the surface temperature of infected areas in mule deer (*Odocoileus hemionus*) infected experimentally with the foot-and-mouth disease virus. In their subjects, it was possible to identify an increase in the maximum temperature in the podal region of 2 °C two days post-infection (*p* = 0.017). Given this initial observation, the authors proposed that detecting thermal body changes may make it possible to identify inflammatory responses that would help diagnose podal and joint pathologies and monitor claudication and pododermatitis in the species mentioned above [13,27,55,82].

Working with Asian elephants (*E. maximus*), Avni-Magen et al. [55] evaluated the sensitivity and specificity of IRT in the foot region to identify inflammatory conditions. The regions evaluated were divided into three groups (one negative, one positive, and one pre-inflammatory). In the group of pre-inflammatory areas, temperatures were 2 °C higher than in the healthy areas. The authors also reported that IRT showed a sensitivity of 89.2% and a specificity of 83.4%, with a predictive value of 99.4%. These results demonstrate the usefulness of this window for this type of inflammatory disorder in large mammals. Similarly, in bird species, Duncan et al. [83] evaluated the foot region of 67 penguins for 15 months, including specimens of king (*Aptenodytes patagonicus*), macaroni (*Eudyptes chrysolophus*), and southern rockhopper penguins (*Eudyptes chrysocome*). They found that when an active lesion was present, the affected area showed a temperature 1.5 °C higher than in inactive or uninjured areas, with a positive correlation between foot and lesion site temperature (rho= 0.94, *p* = 0.0001). In addition, Porter-Blackwell et al. [84] evaluated 10 Falconiformes of the red-tailed (*Buteo jamaicensis*), Harris’ (*Parabuteo unicinctus*), and Swainson’s hawks (*Buteo swainsoni*) species, to study tarsal and plantar thermal responses in limbs with injuries in these areas. They found a significant correlation between the metatarsal and plantar temperatures when abrasive lesions were present (r = 0.81).

At the foot level, IRT has been suggested as an effective method for evaluating thermoregulation processes in other species. Phillips and Sanborn [85], for example, evaluated the thermoregulatory capacity of birds such as the ostrich (*Struthio camelus*), emu (*Dromaius novaehollandiae*), and double-wattled cassowary (*Casuarius casuarius*), species in which the legs constitute a high percentage of the total anatomy and are involved in heat regulation and exchange. It has been reported that the feet and toes of those birds make up 12–17.5% of the total body surface area and that 40% of the metabolic heat they produce is eliminated through these surfaces; thus, they should be considered important sources of heat dissipation. In a study of otters, Kuhn and Meyer [23] found that vasomotor responses in the feet and tail are the main mechanisms for regulating heat loss. The sparsely-furred paws of the Eurasian otter (*L. lutra*) are the only body parts where significant heat loss may occur. When otters are in the water or at low temperatures, vasoconstriction in their skin prevents excessive heat loss, while vasodilation occurs to dissipate excess heat favored, as well, by the interdigital membranes that increase the surface area available for heat exchange.

As a result, in animals where diagnoses of foot or footpad lesions are common, IRT can identify potential lesions that could affect locomotor activity and the quality of life of individuals, as well as the thermoregulatory role of the legs in some species. To date, however, its sensitivity and specificity have only been determined in elephants. Given this scarce evidence, the possibility of validating this window is limited, complicated by the reality that factors such as a generalized inflammatory response may impede precisely identifying the affected areas.

## 6. Limitations on the Use of IRT in Wildlife Species under Human Care

**Figure 11:**
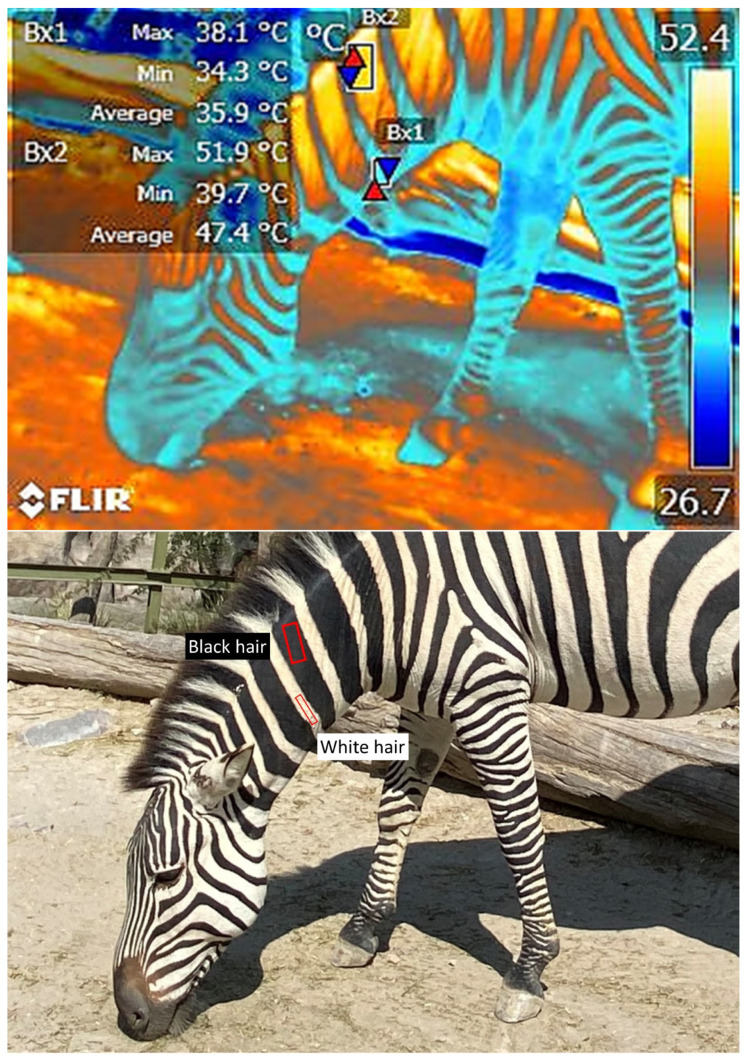
Effect of coat color on the surface temperature of a zebra (*Equus quagga*). It has been suggested that the zebra’s stripes play a role in thermoregulation. IRT images show that the average temperature of the white stripes in the neck is 35.9 °C (Bx1), while the average temperature of the zone with black hair is 47.9 °C (Bx2), a difference of 11 °C. This evidence reaffirms the importance of considering hair color in some species when using IRT since it can influence the amount of solar radiation that may be absorbed. (Radiometric images and photographs by the authors).

Scientific evidence has shown that IRT has clinical and diagnostic applications with zoo animals [64]. However, effectively implementing this tool requires considering factors that can influence recordings and their interpretation. Hilsberg-Merz [1] mentions that the quality and length of an animal’s coat can prevent elements such as mud or dust from interfering with thermographic readings. Other elements to be considered are species-specific thermoregulation mechanisms, morpho-anatomical characteristics such as coat color patterns, hair length, dermal thickness, location of glands, size of ears or appendages such as antlers, and the presence or absence of hair, fur, or feathers [55]. Coat colors and patterns can influence readings and heat loss during short-wave solar radiation interactions. In this case, darker coats have higher absorptivity and generate higher IRT readings, though this only corresponds to higher transmissivity in certain cases. The primary effect is that, as the coat assumes a higher temperature, there is a less favorable thermal gradient for heat exchange through radiation between the epidermis and the coat. Likewise, as Mota Rojas et al. [86] mentioned, hair characteristics such as length and color variation also influence heat loss or gain. Figure 11 shows how the color of the hair in bicolor animals like zebras generates a distinct heat radiation pattern that helps maintain thermostability in these species.

**Figure 12:**
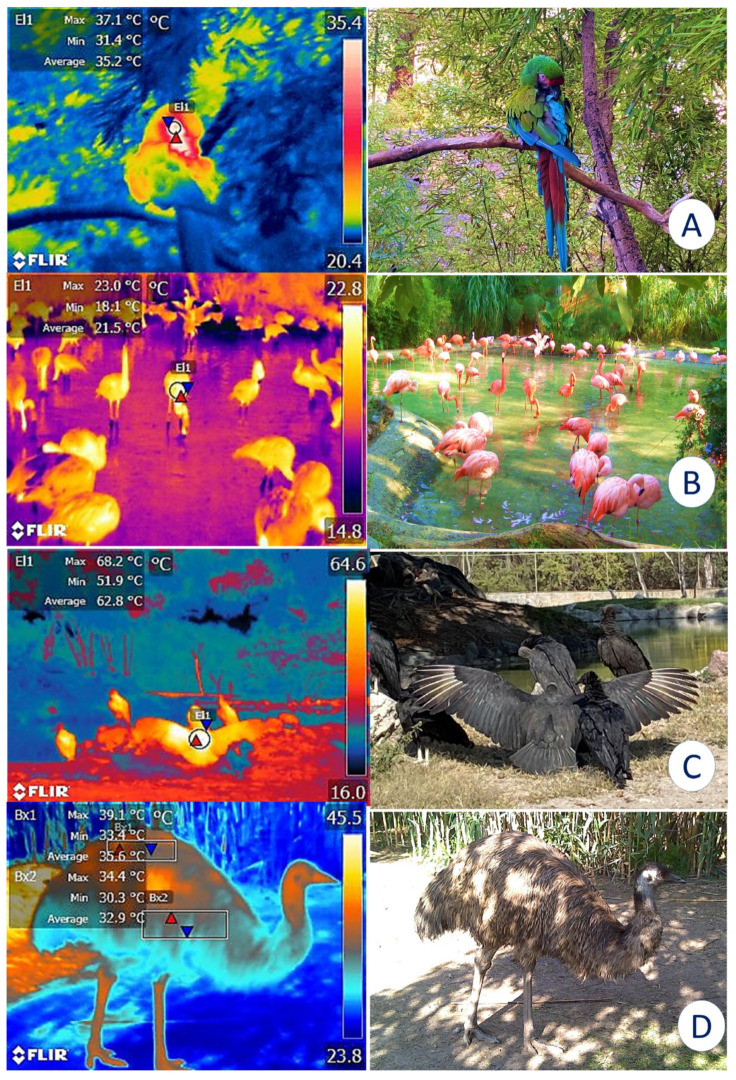
Differences in the thermoregulatory performance of birds with different types of feathers. (**A**). Radiometric image of a military macaw (*Ara militaris*) at rest on a perch. In this species, the ocular window can serve to evaluate thermal responses by detecting average ocular temperatures (El1) (35.2 °C). (**B**). The dorsal region (EL1) of flamingos (*Phoenicopterus ruber*) covered by feathers registered an average value of 21.5 °C despite direct exposition to solar radiation. (**C**). In contrast to the light-colored feathers of the flamingo, the dorsal area (El1) of a black vulture (*Coragyps atratus*) shows an average temperature of 62.8 °C with a maximum value of 68.2 °C. In the digital image of the vulture, a spread-wing posture is observed. This is a thermoregulatory behavior that helps dissipate heat when birds experience temperatures above their thermoneutral range. (**D**). Infrared reading of an emu (*Dromaius novaehollandiae*) where the temperature in the dorsal (Bx1) and ventral regions (Bx2) can be compared, observing an average difference of 6.2 °C. This difference may be attributable to the presence of long feathers that aid in dissipating heat. (Radiometric images and photographs by the authors). Maximum temperature (red triangle), and minimum (blue triangle). Thermal images obtained using a FLIR thermal camera.

Similarly, the presence of feathers in birds and their color and length can alter the body’s ability to dissipate heat [87]. Feathers are known as a thermal insulating mechanism that maintains the temperature of birds within an average value of 40 °C. Therefore, when applying IRT techniques to these species, the role of feathers must be considered to interpret their thermoregulatory capacity accurately [88]. Figure 12 presents thermal images of different bird species to exemplify the importance of this factor.

Despite thermography’s clinical and diagnostic applications, climatic factors that may influence readings, such as exposure to direct solar radiation, precipitation, wind, and humidity, must all be factored in [89,90]. One example of this is a study of large felines in distinct habitats, where the presence of water influenced readings by as much as 1 °C. Findings like those confirm the need to acclimatize the animals for up to two hours before taking radiometric images [21].

The corpus of evidence reviewed suggests that implementing IRT in zoos and conservation centers could serve as a method for selecting and monitoring ideal habitat design, which must meet the needs of each particular species [91]. In practical terms, using IRT with these species could generate information on thermal comfort levels with respect to the characteristics of their microenvironments and help determine the correct features for promoting their welfare and appropriate behaviors.

## 7. Perspectives for Thermography Applied to Zoo Animals, and Other Windows Suggested

Although various windows have been identified for most species of animals in captivity, some studies propose specific anatomical regions for each animal. In the case of capybaras (*Hydrochoerus hydrochaeris*), IRT was used to monitor the health of adult and young animals by taking thermograms of the facial, joints, and head regions. In these animals, the temperature increases registered in the supranasal gland of resting adults made it possible to improve preventive medicine measures with no need for handling or containment [92].

IRT has been used with the genital region of chimpanzees, as well as with the animals’ facial, auricular, and nasal areas, and their hands, neck, and feet, to identify the adaptive behaviors that females have developed to conceal gestation and avoid infanticidal behavior, a common practice of the males in the group. Observations of these females showed that, in contrast to the monitoring of gestation in rhinoceroses, pregnant chimpanzees had similar temperatures to non-pregnant females in the genital tumescence period during the swelling stage, with a maximum difference of approximately 1 °C during the other stages. Their findings led the authors to conclude that in this species, IRT is not a reliable tool for detecting gestation due to the adaptive behaviors of females [57]. In contrast, the vaginal sheath of elephants and rhinoceroses is suggested as an adequate region for detecting stages of the reproductive cycle, such as estrus. Other authors have recorded temperature increases in relation to reproductive behaviors such as mounts by males [1].

**Figure 13:**
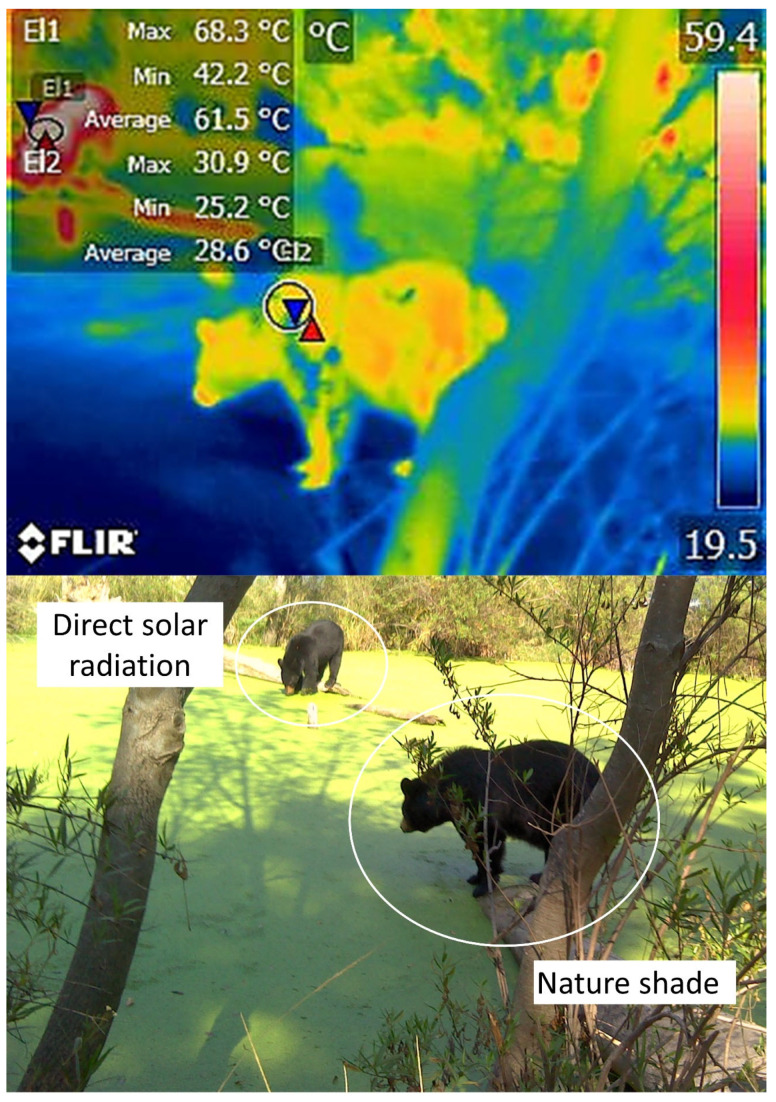
Comparison of the surface temperature in the dorsal area of two American black bears (*Ursus americanus*). Upon assessing the influence of shaded areas on the thermal state of these animals, it is clear that the bear standing under the shade (right side of the thermogram) has an average dorsal superficial temperature of 28.6 °C. In comparison, the individual exposed to direct solar radiation recorded an average value of 61.5 °C. The difference of 32.9 °C between these two animals is an important trait that needs to be considered when designing enclosures for wildlife under human care (Radiometric images and photographs by the authors).

In Malayan sun bears (*Helarctos malayanus*) and polar bears (*Ursus maritimus*) at temperatures of 5–30 °C and 0–20 °C, respectively, the surface temperature of their eyes, snout, face, trunk, shoulder region, inner leg, and outer leg were compared to determine the range of the thermoneutral zone by identifying an absence of thermal exchange on the body surface, a phenomenon that is necessary for both species. It has been suggested that IRT could help assess the thermoneutrality zone in Malayan bears by relating the thermal aspect to autonomic and behavioral responses. The authors found a proportional increase in IRT when ambient temperatures increased in all windows tested except the nasal window. A range of 24–28 °C was registered as ideal for those bears. In other results, when environmental temperatures reached 15 °C (a value below the minimum range), numerous behaviors were altered, including resting, foraging, nesting, social play, physical activity, and standing [93]. The study of polar bearsfound that the test animals showed signs of heat stress at temperatures of 12–19 °C, with temperature increases of 18–24.5 °C in the shoulders and back. Their findings allowed the authors to establish a range in which body and environmental temperatures were close (24–28 °C) and to suggest that the zone of thermoneutrality for this species is limited. Finally, they ascertained that, in polar bears, the extremities and shoulder region are the main areas of heat loss [20]. Thermal images of black bears (*Ursus americanus*) taken by the authors (Figure 13) have proven useful in identifying differences in heat radiation associated with the characteristics of the enclosure. In that case, providing natural shade prevents direct solar radiation from reaching the animals, thus reducing the presentation of heat stress.

In addition, somewhat unconventional applications have been explored in other windows, such as the nasal one. For example, Dunbar et al. [29], evaluated temperature variations in regions such as the dorsal, ocular, and nasal windows in raccoons (*Procyon lotor*) inoculated with the rabies virus. The authors found an increase of 30.4 ± 3.5 °C in the nasal window of the infected animals before the onset of the prodromic phase of the disease (*p* < 0.0001). For this reason, thermographic studies of this kind have been proposed as indicators contributing to both animal and public health.

In light of these findings, future study trends should focus on establishing precise applications for each thermal window according to the characteristics of individual animal species. The objective is to implement non-invasive methods to monitor and optimize thermal responses, health, and environmental conditions of wild animals under human care [9].

## 8. Conclusions

IRT is a tool with potential applications in wild animals under human care. Its non-invasiveness and capacity for remote evaluation are attractive qualities for assessing the health status of wild species with no need for means of chemical or physical restraint, mainly when used in conjunction with other techniques.

In several species of felines, herbivores, birds, canines, and non-human primates, IRT has identified infectious pathologies, inflammatory processes derived from injuries, and physiological states such as gestation. Efforts have also been undertaken to use IRT to understand the emotional states of animals better. However, as we know from experience with humans and domesticated animals, IRT has individual, environmental, and technical limitations that must be evaluated when interpreting the thermal responses of animals.

The importance of implementing these techniques in zoo animals is not limited to animal welfare, though this is a priority for wildlife species under human care. For veterinarians and zookeepers, IRT can also provide information about the conditions of enclosures and how their design can alter the thermoregulatory mechanisms of diverse species by providing environments that better satisfy their biological needs, prevent heat stress, and facilitate preventive health monitoring. Another benefit of applying IRT in zoos is the potential contributions that findings could make to our scientific knowledge of wild animals in captivity that would be impossible to obtain in wild conditions. Finally, the information obtained by utilizing IRT in zoos can be applied to wild populations. Approaches of this kind would support the role of zoos as wildlife research and conservation centers, where techniques such as IRT are available to evaluate, objectively and non-invasively, the daily activities of animals, aid in preserving positive mental states, and, in that way, ensure proper clinical, behavioral, physiological, habitat, and emotional welfare.

## Data Availability

Not applicable.
